# Cavity-Enhanced 2D Material Quantum Emitters Deterministically Integrated with Silicon Nitride Microresonators

**DOI:** 10.1021/acs.nanolett.2c03151

**Published:** 2022-11-01

**Authors:** K. Parto, S. I. Azzam, N. Lewis, S. D. Patel, S. Umezawa, K. Watanabe, T. Taniguchi, G. Moody

**Affiliations:** † Electrical and Computer Engineering Department, University of California, Santa Barbara, California93106, United States; ‡ California Nanosystems Institute, University of California, Santa Barbara, California93106, United States; § Research Center for Functional Materials, National Institute for Materials Science, 1-1 Namiki, Tsukuba305-0044, Japan; ∥ International Center for Materials Nanoarchitectures, National Institute for Materials Science, 1-1 Namiki, Tsukuba305-0044, Japan

**Keywords:** quantum emitter, microresonator, silicon nitride, Purcell enhancement, hexagonal boron nitride

## Abstract

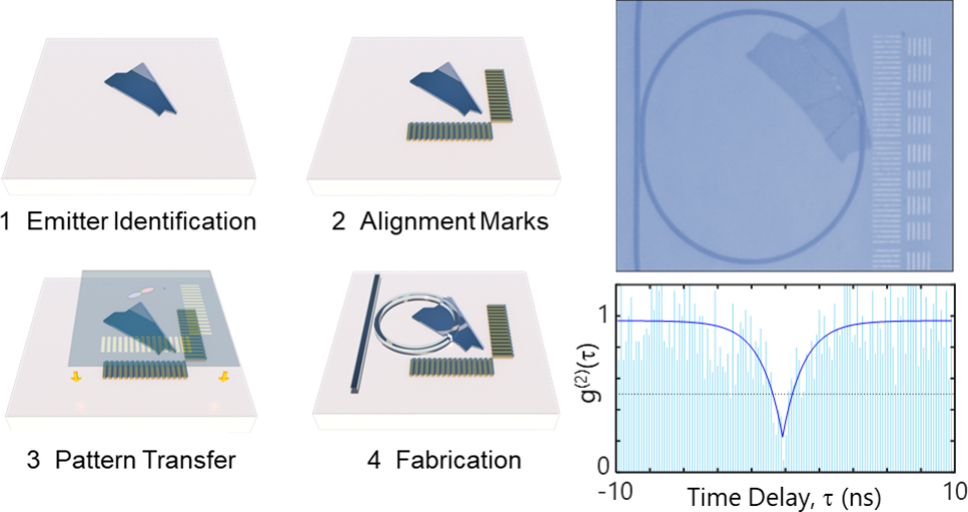

Optically active defects in 2D materials, such as hexagonal boron nitride (hBN) and transition-metal dichalcogenides (TMDs), are an attractive class of single-photon emitters with high brightness, operation up to room temperature, site-specific engineering of emitter arrays with strain and irradiation techniques, and tunability with external electric fields. In this work, we demonstrate a novel approach to precisely align and embed hBN and TMDs within background-free silicon nitride microring resonators. Through the Purcell effect, high-purity hBN emitters exhibit a cavity-enhanced spectral coupling efficiency of up to 46% at room temperature, exceeding the theoretical limit (up to 40%) for cavity-free waveguide-emitter coupling and demonstrating nearly a 1 order of magnitude improvement over previous work. The devices are fabricated with a CMOS-compatible process and exhibit no degradation of the 2D material optical properties, robustness to thermal annealing, and 100 nm positioning accuracy of quantum emitters within single-mode waveguides, opening a path for scalable quantum photonic chips with on-demand single-photon sources.

Solid-state single-quantum emitters (SQEs) integrated with chip-scale photonic circuitry are key building blocks for quantum information technologies, including linear optical computing, cluster state generation, quantum key distribution, and quantum random number generation.^1−3^ Numerous SQEs capable of high-purity single-photon emission have been discovered in several materials, including semiconductor quantum dots,^2^ diamond,^4^ silicon nitride,^5^ and two-dimensional materials.^6^ The integration of SQEs with CMOS-compatible photonics would address a longstanding need for combining the manufacturability and scalability inherent to silicon-based photonics with materials that host high-quality SQEs. Heterogeneous integration techniques have led to successful demonstrations at cryogenic temperatures, including arrays of diamond SQEs coupled to aluminum nitride photonic integrated circuits (PICs)^7^ and self-assembled quantum dots integrated with silicon nitride.^8,9^ Yet, scalable strategies for the integration of SQEs with silicon-based PICs have not yet been demonstrated. Four key requirements are necessary to address this challenge: (1) a host material with high purity, high indistinguishability (*V*), and bright emitters, (2) the ability to integrate the SQE host material with the PIC platform without degrading the optical properties, (3) control of the emission wavelength and precise alignment within low-loss and background-free single-mode waveguide structures, and (4) integration with microresonators to enable Purcell enhancement of single-photon extraction efficiency (η) and indistinguishability (maximizing η × *V*) into a single optical mode.

Of the SQE platforms, defect-type emitters in 2D materials^6,10−12^ have emerged as an attractive approach for engineering single-photon sources. SQEs have been identified in several 2D materials spanning ultraviolet to telecommunications wavelengths, including hexagonal boron nitride (hBN),^13−15^ transition-metal dichalcogenides (TMDs),^16−23^ and heterostructures.^23,24^ In hBN and WSe_2_, >10 MHz emission rates^25,26^ and 95% single-photon purity have been reported. Through strain and defect engineering, emitters can be aligned into arrays,^27,28^ and nanophotonic integration further enhances their brightness.^29,30^ SQEs in hBN are particularly appealing due to a 5.7 eV band gap, which enables room-temperature generation of single photons with up to 93% purity.^25^ The observation of mechanically decoupled emitters in hBN with transform-limited line widths shows a promising path toward the emission of indistinguishable photons at cryogenic temperatures.^31,32^ Both indistinguishability and on-chip brightness are important, and thus it is critical to maximize the coupling efficiency–indistinguishability product (η × *V*) while maintaining a high purity of the emitters. While previous reports have shown 2D emitters coupled to fiber and planar cavities for off-chip collection,^33−35^ 2D emitters integrated with cavities for on-chip collection into waveguides have not been demonstrated yet. Previous on-chip integration strategies that have placed hBN and WSe_2_ directly on waveguides suffer from low coupling efficiencies (a few percent) due to the random position and dipole orientation of the SQEs. Even for perfectly positioned and aligned emitters in a single-mode waveguide, the finite mode confinement limits the maximum simulated coupling efficiency^36^ up to ∼40% (∼20%) for emitters embedded at the center of (below) the waveguide (see the Supporting Information for details of modeling). Furthermore, commonly used stochiometric silicon nitride, which is an excellent platform for 2DMs due to its low propagation loss,^37^ large refractive index,^38^ and wide transparency window that spans all 2D SQEs (Figure 1a), has a strong fluorescence^39^ that reduces the single-photon purity of integrated SQEs, further complicating their optimal on-chip integration.

**Figure 1:**
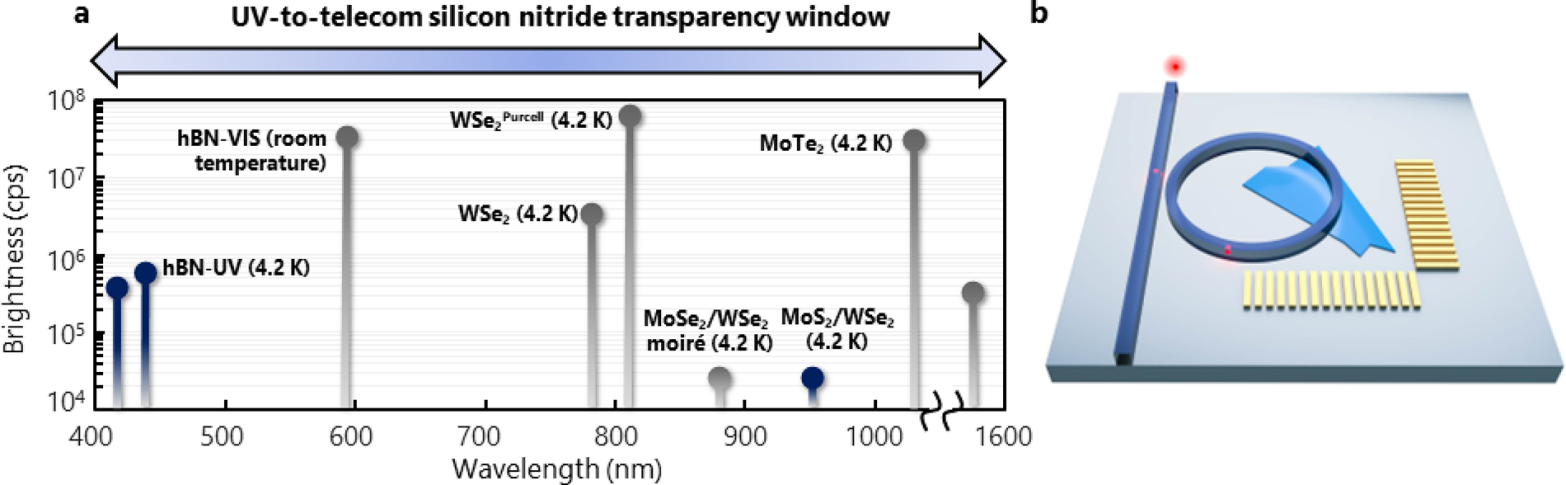
Universal platform for precision integration of 2D quantum emitters in silicon nitride photonics. (a) The family of 2D materials, including hBN emitters at UV^15,57^ and visible^25^ wavelengths, TMD monolayers,^23,26,52^ and TMD heterostructures,^24,58^ exhibiting a rich spectrum of quantum emitters spanning the ultraviolet-to-telecommunications wavelength transparency window of silicon nitride photonics. The height of each bar indicates the reported intensity of the photoluminescence from the class of emitters (the gray data points are brightness-corrected for the objective extraction efficiency, whereas the blue data points are reported at the detector). (b) Concept for the deterministic integration of a 2D material quantum emitter embedded within a silicon nitride microresonator with its electric dipole aligned to maximize overlap with the cavity modes for large Purcell enhancement and coupling efficiency.

Here, we demonstrate a novel method for efficient on-chip coupling by integrating 2D SQEs with microring resonators using a CMOS-compatible process. Our approach is universal in that it enables the deterministic integration of SQEs with low autofluorescence and single-mode silicon nitride photonic circuits with precise control over the emitter placement and dipole orientation within the waveguiding structures—both of which are critical for achieving efficient coupling. We demonstrate this approach by integrating hBN SQEs generating single photons at room temperature with waveguide-coupled microring resonators (Figure 1b). Emitter–cavity coupling of up to 46% is measured, which requires only a modest Purcell factor of 0.86 ± 0.15 to surpass the waveguide coupling efficiency in prior studies by nearly 1 order of magnitude.^36,39−41^ We demonstrate the universality of the approach by also coupling exciton emission from embedded WS_2_, achieving >63% efficiency with a Purcell factor of 1.44 ± 0.25. We present various emitter–microresonator designs, coupling schemes, and metrics that provide a roadmap for the integration of SQEs spanning the UV to telecommunications wavelength regimes. Guided by a semiclassical cavity–emitter model, routes toward achieving high-purity, high-indistinguishability (η × *V*) single-photon emission from a variety of 2D material emitters are proposed, paving the way for enabling scalable and manufacturable integrated quantum photonics with on-demand sources in silicon nitride. Standard plasma-enhanced chemical vapor deposition (PECVD) of stoichiometric Si_3_N_4_ suffers from significant background fluorescence^5,39,42−44^ that overlaps with the emission from many 2D materials, including hBN and TMDs. To address these challenges, we extend previous developments of nitrogen-rich silicon nitride (SiN) that eliminate the background fluorescence. Careful tuning of the PECVD RF power, voltage bias, and silane to ammonia ratio (*R*) allows for the deposition of high-quality silicon nitride with negligible background fluorescence and a high refractive index, without damaging the underlying 2D materials.

Figure 2 illustrates that, for decreasing *R*, the fluorescence is quenched with only a moderate reduction in the refractive index; however, creating SQEs in hBN is typically achieved^45^ through rapid thermal annealing up to 1100 °C. In previous studies, annealing has introduced or activated defects, which enhances the fluorescence background even in nitrogen-rich films.^39^ By preconditioning the annealing chamber with an optimized oxygen/nitrogen environment, we find that the defect band remains absent for temperatures at least as high as 1000 °C. This points to extrinsic defects being introduced from the chamber as one of the primary sources of the fluorescence. Figure 2c illustrates the results from this process. A room-temperature photoluminescence spectrum is shown in Figure 2c from a representative hBN emitter in a flake under a 100 nm thick low-stress stoichiometric Si_3_N_4_ film. Nearly 50% of the emission at the zero-phonon-line (ZPL) wavelength of the emitter near 540 nm arises from the Si_3_N_4_ emission. Using the new thin-film deposition procedure, we observe emission from hBN emitters with negligible background from the SiN, as shown in Figure 2d for bare hBN (red curve) and the same hBN after growth and annealing of 100 nm SiN (blue curve).

**Figure 2:**
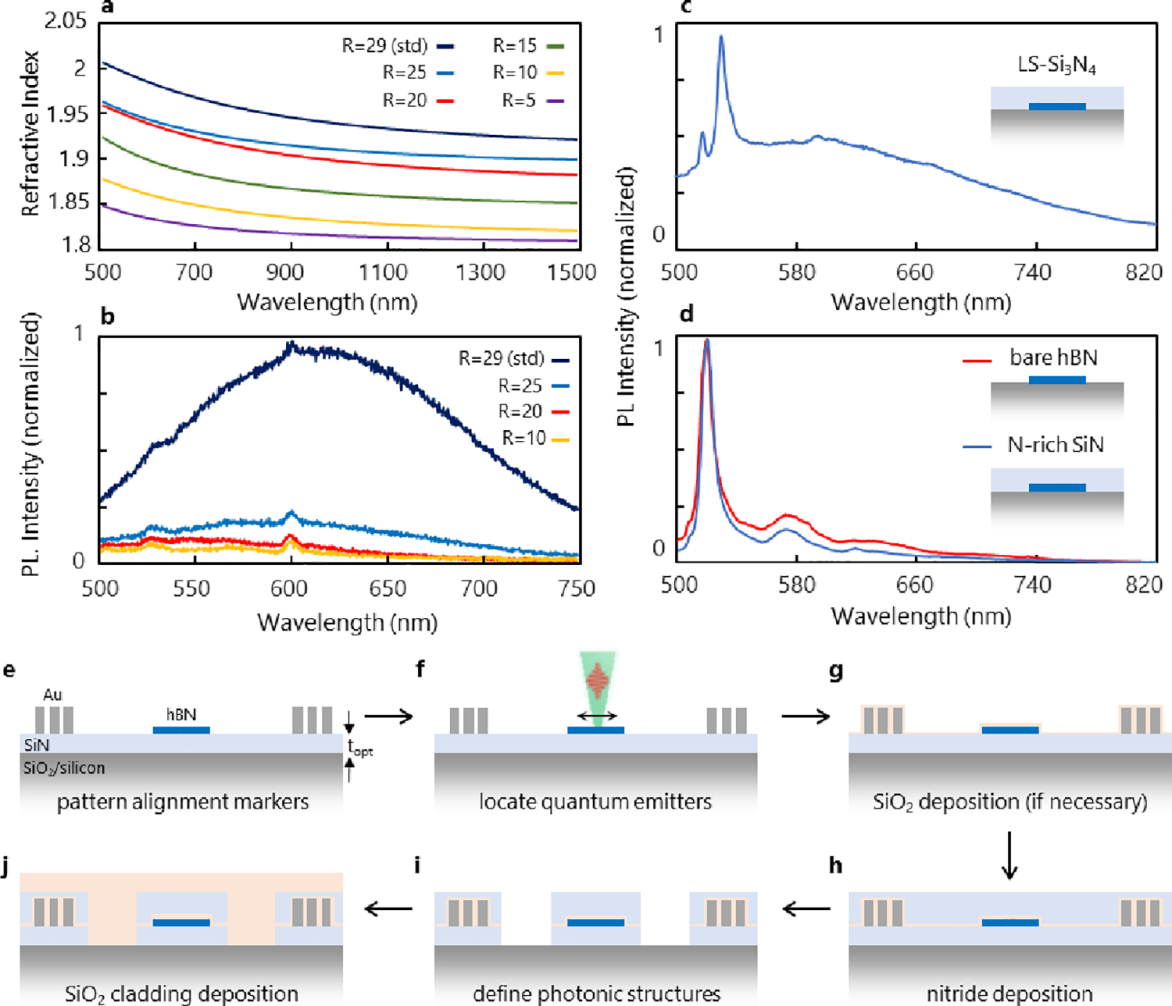
Site-specific and momentum-aligned integration of hBN SQEs to low-emission silicon nitride. (a) Refractive index of silicon nitride as a function of decreasing silane/ammonia ratio, *R*. (b) Background emission of silicon nitride as a function of *R*. At *R* = 20, the background emission is sufficiently suppressed for high-purity measurements of hBN SQEs. (c) PL spectrum of an hBN SQE covered with 100 nm of low-stress stochiometric PECVD Si_3_N_4_. (d) PL spectra of an hBN SQE before (red) and after (blue) 100 nm deposition of nitrogen-rich nonstochiometric PECVD SiN and 1000 °C rapid thermal annealing, acquired at the same excitation power and integration time as in (c). (e) To position the flake with respect to the photonic structure, thin hBN flakes are exfoliated on PECVD SiN films with an optional thickness (*t*_opt_) that is either equal to all or half of the designed thickness of the waveguide for top or embedded flakes, respectively. Alternatively, to position the flake on the bottom of the waveguide, hBN can be exfoliated directly on the SiO_2_ substrate. Gold alignment markers are patterned with electron-beam lithography in close proximity to the flake. (f) Position and dipole orientation of quantum emitters determined by high numerical-aperture polarization-resolved microscopy and raster scanning of the sample. (g) Thin-film PECVD SiO_2_ for protecting TMD flakes from damage during SiN PECVD. (h) Deposition of remaining SiN, if necessary, to complete the photonic layer. (i) Electron-beam lithography and ICP-RIE etching used to define the photonic circuits. (j) Final PECVD SiO_2_ for the cladding layer.

The fabrication process for deterministically embedding 2D flakes within SiN photonic structures is illustrated in Figure 2e–h, which enables the fabrication of photonic devices aligned to emitters with 100 nm precision (see the Supporting Information). This procedure, combined with the ability to deposit and anneal SiN on top of the 2D emitters without damaging them, enables flakes to be integrated throughout the cross-sectional profile of the structures. Figure 3c shows the theoretical coupling efficiency of an emitter with perfect polarization alignment integrated with a single-mode SiN waveguide at different heights for three types of 2D materials. The coupling efficiency of the radiated field into the waveguide mode, normalized to the total radiated field, is defined as β, where β = 1 corresponds to 100% emission into the waveguide mode. Intuitively, the greatest mode overlap occurs when flakes are embedded in the center of the waveguide; however, care must be taken to avoid etched hBN edges within a few hundred nanometers of the emitter, which can introduce edge states and lead to optical dephasing and spectral diffusion. Thus, we also explored the integration of hBN underneath the waveguide in which the hBN flake is not exposed to any etched surfaces. For this configuration, a theoretical coupling efficiency of β = 20% (Figure 2c) is expected. In practice, the measured waveguide coupling efficiency, however, is typically limited to ∼1–3% primarily due to poor emitter-mode overlap and dipole misalignment.^36,40,41^

**Figure 3:**
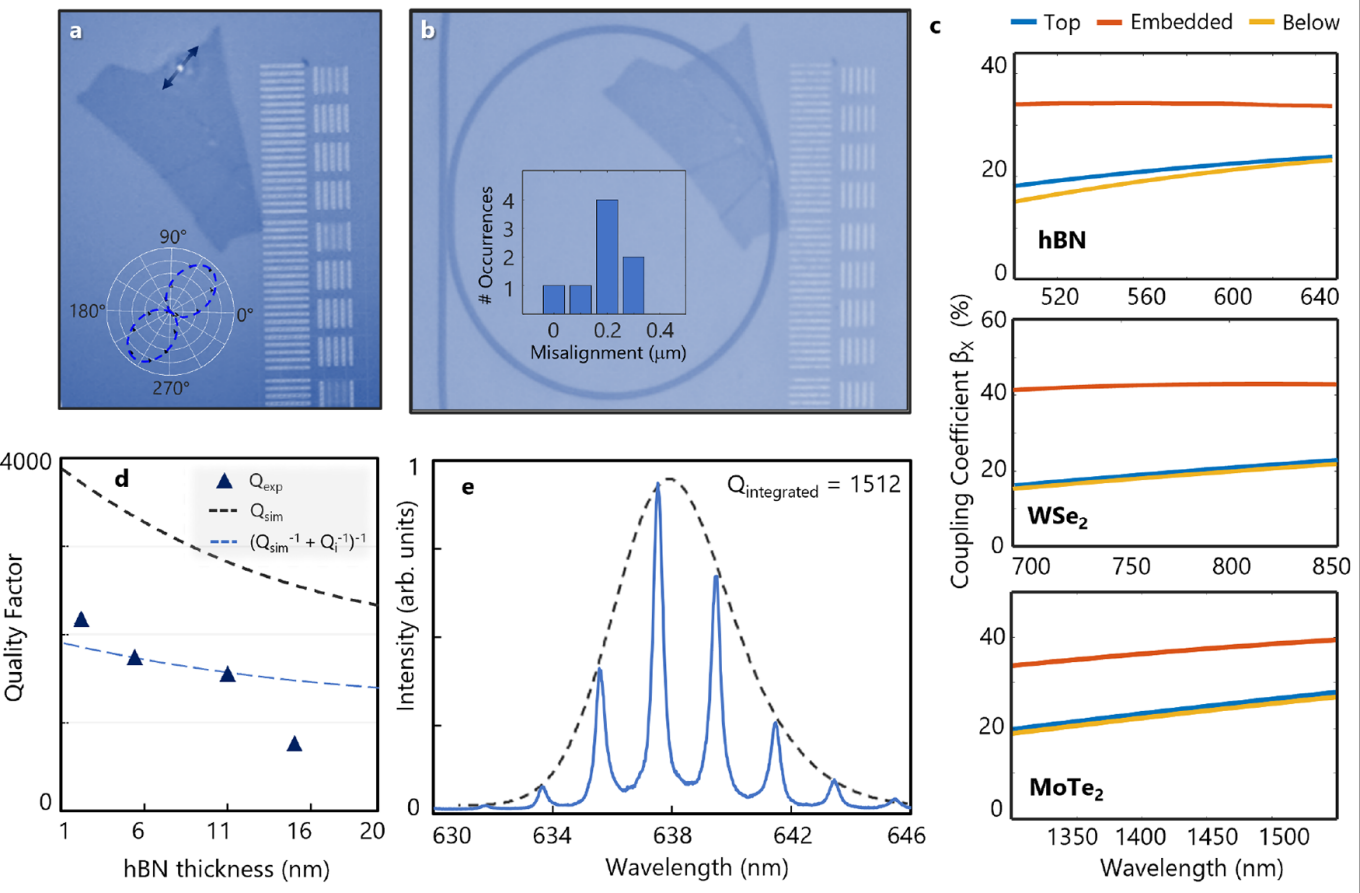
Integration of hBN SQE and microresonator characterization. (a) Optical image of an SQE in an hBN flake near positioning markers. The SQE location is denoted by the bright white dot, which corresponds to the dimmed excitation laser spot. The inset plot depicts the emission dipole orientation of the emitter. (b) Fabrication of a complete device with an hBN emitter integrated and dipole-aligned to a microring resonator with 100 nm precision. The inset plot shows the distribution of the SQE positioning over several trials. (c) Simulated waveguide–emitter coupling efficiency (β) for 2D quantum emitters on top, within, or below the straight waveguide. A maximum of up to 40% coupling efficiency (summed over both waveguide propagation directions) is possible for each type of emitter. (d) Theoretical (lines) and experimental (points) resonator quality factor as a function of the integrated hBN flake thickness. It is assumed the flake covers 25% of the ring in the simulations. The black dashed line represents the theoretical simulations, which do not take into account the intrinsic loss due to the flake and absorption in the guiding medium. The blue dashed line represents the theoretical simulations corrected with the experimental intrinsic quality factor. (e) Characterization of a ring with an integrated hBN flake. A broad-band superluminescent diode (centered roughly at 638 nm, dashed line) is coupled to the output port of the waveguide. Scattered light from the ring (solid line) is collected using a 0.9 NA objective. A free-spectral range (FSR) of 2.1 nm and a loaded quality factor (*Q*) of 1512 are measured with an embedded flake.

Alternatively, β can be enhanced relative to waveguides by integrating the emitter within an optical cavity. For a cavity-coupled emitter, its radiative decay rate is resonantly enhanced and becomes , where Γ_0_ is the radiative decay rate in the absence of the cavity and *F*Γ_0_ is the radiative enhancement due to the cavity.^46,47^ This enhancement can be quantified through the Purcell factor  where *n*_cav_, *Q*, and *V* are the refractive index, quality factor, and cavity mode volume, respectively. For a cavity-coupled emitter,^47,48^ β can be expressed in terms of the Purcell factor as . As we show experimentally below, even for *F* ≈ 1, the on-chip SQE emission can be significantly enhanced relative to an emitter coupled to a waveguide.

The Purcell factor is typically defined in the “good-emitter” regime in which the cavity line width κ is larger than the SQE line width γ; however, in many instances, including hBN emitters at room temperature, phonon-induced dephasing broadens the ZPL width beyond the radiative limit, and the cavity-coupled system is found in the “bad-emitter” regime, i.e. γ > κ, where only a portion of the ZPL couples into the cavity. This reduces the traditional Purcell factor to *F*κ/γ, where κ/γ heuristically represents the ratio of the radiated power from the SQE that overlaps with the cavity mode. In the bad-emitter regime, a wavelength-dependent Purcell factor and coupling efficiency can be defined in terms of the spectral power of the emitter, *F*_s_(λ) = *I*_cav_(λ)/*I*_fb_(λ) and , where *I*_cav_(λ) and *I*_fb_(λ) are the spectral intensities of the emitter into the cavity mode and into free-space, respectively. In effect, this negates the κ/γ factor and allows for the enhancement of the portion of the ZPL that is resonantly coupled to the cavity to be quantified regardless of the emitter–cavity regime.^49^ Whether in the good- or bad-emitter regime,  specifies the cavity enhancement at the emission wavelength λ_0_, while integration over λ determines the total β.

We chose a racetrack resonator configuration with a 100 nm thick and 600 nm wide cross section, a 3 μm long coupler region that results in a free spectral range (FSR) of 2 nm, and a mode volume of  at the resonance of interest around 610 nm (see Figure 3a,b). The simulated quality factor is 7000, which is comparable to the cryogenic line width of hBN emitters observed in our samples. Light is coupled into/out of the waveguide via end-coupling between a single-mode fiber and a tapered waveguide for mode matching. We first characterized resonators without 2D materials to establish a baseline for our quality factor, which we can write as , where *Q*_i_ corresponds to the bare resonator, *Q*_c_ corresponds to the coupling to the waveguide, and *Q*_sc_ arises from scattering from an integrated 2D flake. Measurements from 10 nominally identical resonators from three different fabrication runs yield an average *Q*_i_ = 3560 and *Q*_c_ = 9700, indicating a slightly lower *Q* value than in our simulations likely due to etched sidewall roughness and a larger waveguide–resonator coupling gap.

The impact of integrated hBN flakes on the resonator *Q* is also examined. With increasing flake thickness, *Q* decreases, which matches our simulations (Figure 3d). While hBN has a refractive index similar to that of SiN, light scattering at the SiN–hBN interfaces, which has a more pronounced effect for thicker flakes, dominates the loss and reduction of *Q* in our simulations. Experimentally, for flakes with a thickness exceeding 30 nm, *Q* decreases by 1 order of magnitude. Given that hBN emitters tend to have narrower line width and brighter emission in thin but multilayer flakes, this result confirms an important design tradeoff for resonator integration. We found that emitters with line widths as narrow as 3–4 nm at room temperature are routinely identified in ∼15 nm thick flakes. Figure 3e shows the spectrum of the microresonator with an hBN flake integrated below the ring, exhibiting a loaded *Q* = 1512.

Figure 4a shows the photoluminescence (PL) spectrum of a representative hBN emitter after the complete device fabrication using top-down excitation and collection at room temperature. As demonstrated in Figures 2d and 4a, the integration and fabrication do not degrade the quality of the emitter and we retain the background-free emission. The photon antibunching behavior verifying single-photon emission is illustrated in Figure 4b in which  (0.18 with background correction). We next excite the emitter using a 0.9 NA objective from above the resonator, and emission into the waveguide is collected into a single-mode fiber and sent to a spectrometer and charge-coupled-device camera. Figure 4c shows the integrated hBN SQE with the ZPL emission centered near 610 nm. The solid red line is emission collected from above the emitter, whereas the solid blue line is emission collected from the waveguide, which shows the resonator modes clearly imprinted on the ZPL emission spectrum separated by an FSR of ∼2 nm. A similar response is observed on the room-temperature exciton emission from bilayer WS_2_ as shown in Figure 4d, demonstrating the universality of the approach for 2D material integration.

**Figure 4:**
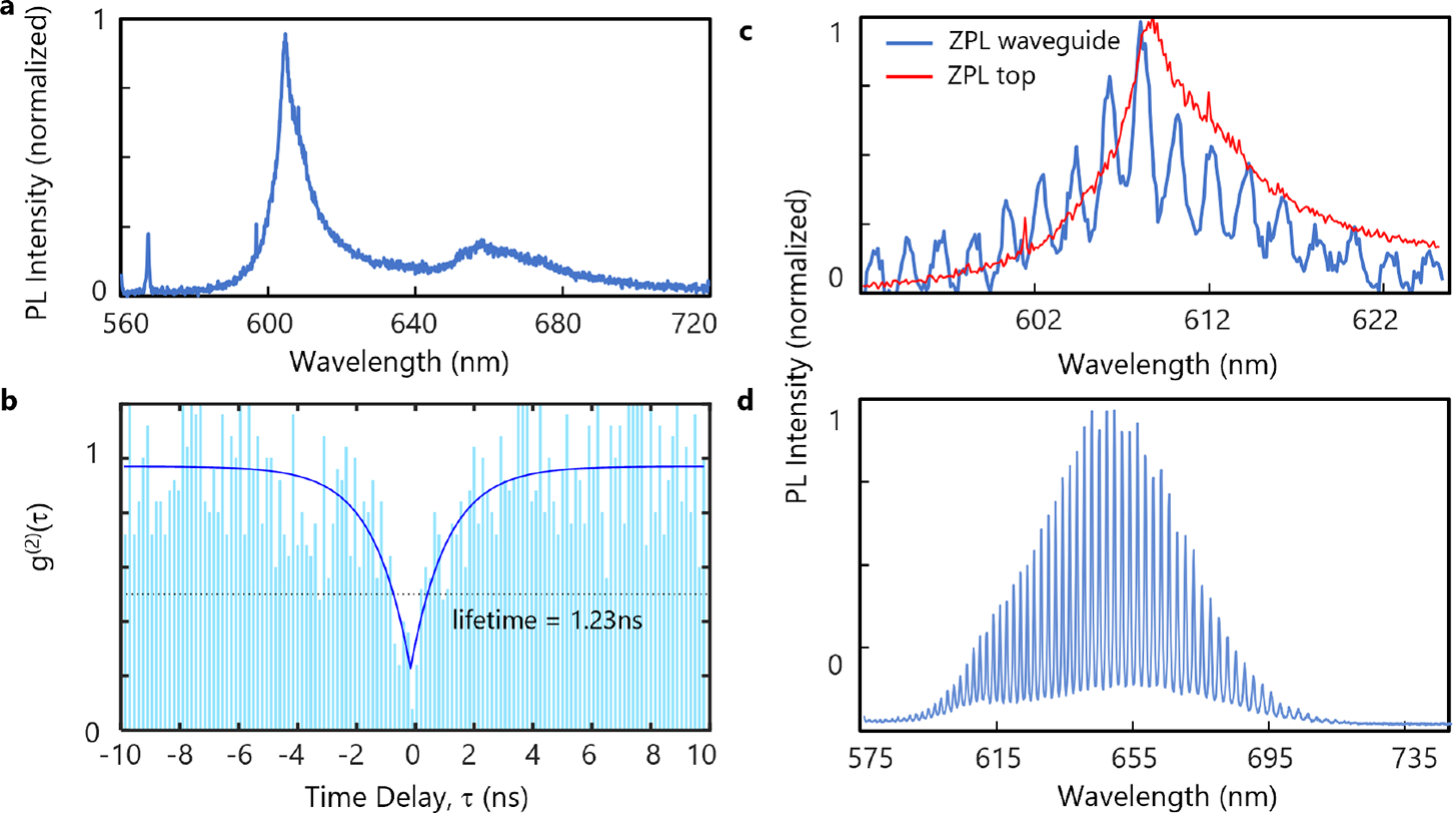
Microresonator-integrated hBN quantum emitter with high coupling and Purcell enhancement. (a) PL spectrum of an hBN SQE acquired with top-down excitation and collection after microresonator integration. The emitter properties are preserved after the fabrication process. (b) Second-order autocorrelation measurement demonstrating 78% purity and a lifetime of 1.23 ns (82% purity when background-corrected). The data are raw with no correction for the background or detector dark counts. (c) ZPL of the emitter observed from the output port of the waveguide using an aligned fiber array (blue line) and from the top collection (red line). A factor of 10 reduction of the ZPL line width is observed (from 7.2 nm down to 0.72 nm), as expected from the bandwidth of the microresonator. The peak intensity of the ZPL is misaligned from the nearest cavity resonance by ∼0.35 nm. (d) Excitonic response of a bilayer WS_2_ collected from the waveguide port. The modes of the microresonator are clearly visible, demonstrating a quality factor of up to 2400.

To extract the spectral Purcell enhancement *F*_s_(λ) and the spectral coupling efficiency β_s_(λ), we follow a procedure previously reported for SQEs.^36,40,46^ Accounting for the optical loss in our system, the effective Purcell enhancement at the ZPL peak wavelength can be expressed as
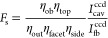
1where η_ob_, η_top_, η_out_, η_facet_, η_side_, , and  are respectively the portion of the total light collected by the top objective, efficiency of the top collection path, microring out-coupling efficiency, facet coupling efficiency, side path collection efficiency, spectral intensity measured on the CCD at the ZPL wavelength from the top objective, and spectral intensity measured from the waveguide output port. From this analysis, we determine a spectral Purcell factor of up to 0.86 ± 0.15 corresponding to β_s_ = 46 ± 4% at the ZPL resonance (β = 28 ± 4% integrated across the entire spectrum). Deviation from the theoretical estimate of the effective Purcell factor for this system (equal to 1.7) can be attributed to small misalignment and dipole orientation inaccuracies. Importantly, even though *F*_s_ is close to unity, this results in nearly half of the emission now coupled into the cavity mode. This is best reflected in β_s_ of the cavity-coupled system. Here, the lower bound of our measured β_s_ exceeds the maximum theoretical coupling efficiency into a waveguide of the same configuration (∼20%) as shown in Figure 2). For this emitter, we measure a 13% reduction of the lifetime after integration, qualitatively consistent with our measured Purcell enhancement; however, we did not rely on lifetime measurements to estimate the Purcell factor due to ambiguities in the quantum yield, nonradiative processes, and whether these are affected by fabrication (see the Supporting Information). Similarly for the integrated WS_2_, β_s_ = 63 ± 4% is obtained, amounting to a spectral Purcell factor of *F*_s_ = 1.44 ± 0.25. The higher measured Purcell factor for WS_2_ is due to the fact that it is thinner and thus does not significantly alter the loaded quality factor of the resonator.

An important figure of merit of an emitter–cavity system is the PIC efficiency–indistinguishability product η × *V*. In the good-emitter regime, the cavity not only provides enhancement in coupling efficiency but also broadens the natural line width to increase the indistinguishability. Total PIC efficiency can be expressed as η = η_qe_ × β × η_out_, where η_qe_ is the quantum efficiency of the emitter and η_out_ is the extraction efficiency of the coupled light in the cavity into the bus waveguide.^46,50^ Maximizing η × *V* is a multivariable problem because the individual components of efficiency and indistinguishability cannot be adjusted independently. For instance, while a high *Q* results in a larger β and *V*, for large *Q*, the cavity–emitter system can enter the bad-emitter regime where only a portion of the ZPL will couple into the microring resonator and η will begin to decrease. Generally, the line width of the emitter sets a practical upper bound for the loaded *Q*. While this can imply that SQEs with the narrowest line width are more suitable for cavity integration, the SQE quantum efficiency η_qe_ also plays an important role in the emitter–cavity design. For instance, η_qe_ for WSe_2_ is estimated^28,29^ to be only ∼1–5% compared to up to 87% reported for hBN.^51^ Therefore, to optimize the cavity design with high η × *V* for different 2D emitters, a holistic approach must be considered.

As shown in Figure 5, we explore the performance of a 2D SQE–cavity system using solutions to a modified Jaynes–Cummings Hamiltonian^49,52^ for the state of the art 2D SQEs interacting with a cavity (see the Supporting Information). Figure 5a shows η × *V* as a function of mode volume and the microresonator quality factor *Q* for near-transform-limited mechanically decoupled hBN emitters at cryogenic temperatures.^31,32^ The vertical dashed line sets the boundary of the bad-emitter regime, in which the total quality factor exceeds the emitter quality factor *Q*_e_ determined from its line width; as *Q*_c_ becomes larger than *Q*_e_, only a portion of the emitter couples to the cavity mode. The horizontal dashed line in Figure 5a indicates the minimum mode volume for our PICs (30(λ/*n*)^3^), and slices along this line are shown in Figure 5b for hBN and TMD emitters. For *Q* ≈ 16000, an η × *V* value of up to 90% is possible with existing hBN emitters at cryogenic temperatures. For *Q* exceeding *Q*_e_, the system enters the bad-emitter regime and η × *V* begins to decrease.

**Figure 5:**
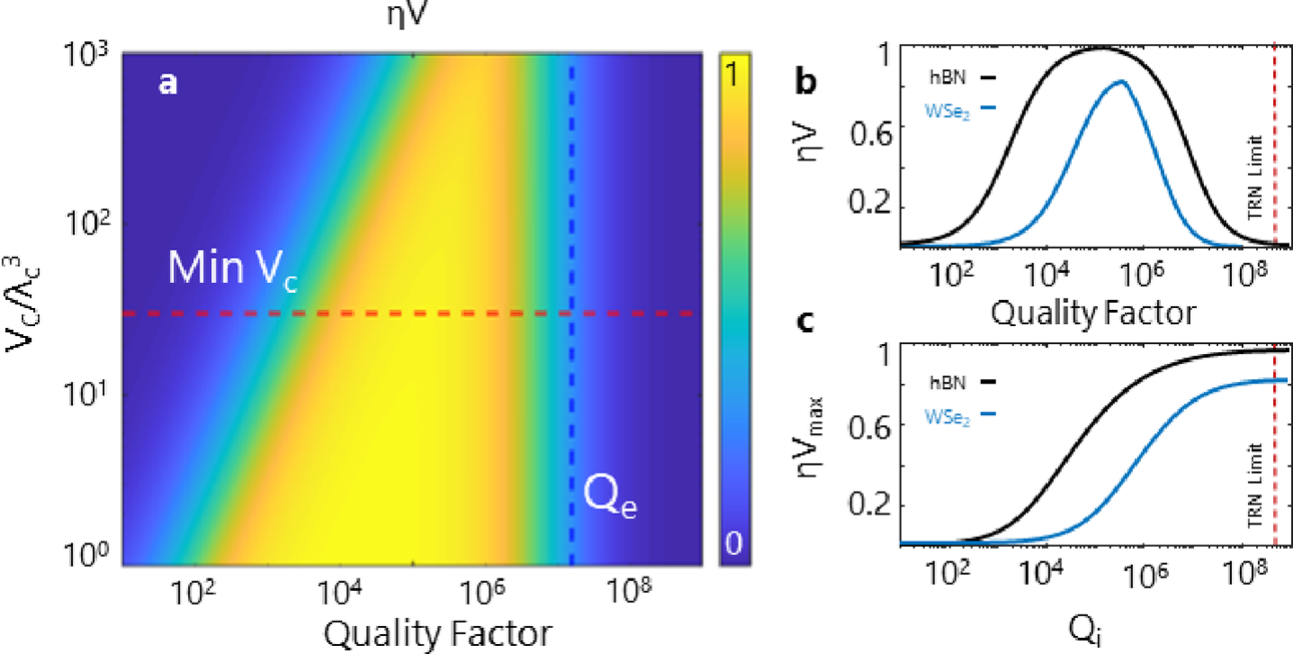
Projected performance of state of the art 2D material quantum emitters in a SiN heterogeneous platform. (a) Mode volume versus coupling quality factor *Q*_c_ for hBN visible emitters (mechanically decoupled near-transform limited line widths^32^). The red dashed line demonstrates the mode volume achieved in our SiN microresonators. The blue dashed line demonstrates the intrinsic quality factor *Q*_e_ of the emitter determined from its line width. (b) Total system efficiency–indistinguishability figure of merit η × *V* as a function of loaded *Q* at the minimum achievable mode volume for hBN and WSe_2_ emitters in the visible wavelength. The intrinsic line width and total dephasing rate are taken from previous resonant fluorescence studies to be 50 and 150 MHz (for hBN^32^) and 100 MHz and 2 GHz (for WSe_2_ monolayers^59^), respectively. (c) Maximum figure of merit η_max_ × *V* achievable for each class of emitters as a function of the intrinsic quality factor of the SiN platform.

Purcell enhancement can compensate for intrinsic low quantum efficiency and indistinguishability of some emitter types, such as WSe_2_, provided loaded *Q* > 10^5^ is reached. Such *Q* values are orders of magnitude below the fundamental thermorefractive noise limit for SiN;^53^ however, further optimization of nonstochiometric growth, side-wall roughness, and engineering emitters in monolayer flakes is required to further improve the quality factors. On the other hand, integration of emitters with high quantum efficiency, such as hBN, can be realized at lower *Q*. Figure 5c shows the maximum attainable η × *V* for each class of the emitters as a function of the intrinsic quality factor of the platform. As the intrinsic *Q* approaches 10^6^, which is already achievable for different SiN waveguide aspect ratios, it is possible to integrate 2D quantum emitters with η × *V* exceeding 80%. These values are competitive with some of the best alternative materials for integrated single-photon emitters, such as self-assembled InAs quantum dots (ranging from η × *V* = 3% for dots emitting on-chip^54^ to up to 78% for dots in a nanopillar cavity^55^) and silicon vacancy centers in diamond^56^ (modeling using the Markovian master equation with dissipative dynamics places η × *V* near ∼80%).

Taken together, our simulations and experiments provide a straightforward approach for deterministically aligning and orienting SQEs in 2D materials to microresonators with a route toward high coupling efficiency. Already this approach achieves 46% coupling efficiency into the resonator at the emitter ZPL resonance, which is 1 order of magnitude higher than waveguide coupling for hBN. A systemwide efficiency approaching 10% can be attained in the near term with modest improvements to the microresonator *Q* and its design for overcoupling. The platform and methods developed in this work can serve as a crucial advancement toward future demonstrations of on-chip 2D quantum emitter integration with high extraction efficiency, brightness, and indistinguishability. In the near future, by exploring SiN microresonators embedded with other SQEs with narrow line widths, such as WSe_2_ and MoTe_2_, new opportunities exist for on-demand and site-controlled SQEs with silicon-based photonics for chip-scale quantum information applications.

## Data Availability

The data that support the findings in this study are available from the corresponding author upon reasonable request.

## Supporting Information Available

Details of the alignment procedure, fabrication methods, numerical modeling of the microresonators, and analytical modeling of the emitter–cavity coupling (PDF)
