# Supplementary material for: Cavity-Enhanced 2D Material Quantum Emitters Deterministically Integrated with Silicon Nitride Microresonators
Source: Nano Lett. 2022 Nov 1;22(23):9748–56. doi: 10.1021/acs.nanolett.2c03151 (PMC9756340; doi:10.1021/acs.nanolett.2c03151)
Supplement: Supplementary file 1 — nl2c03151_si_001.pdf [file nl2c03151_si_001.pdf]

Cavity-Enhanced 2D Material Quantum Emitters Deterministically Integrated with Silicon Nitride Microresonators

K. Parto,[1,a)] S. I. Azzam,[1,2,a)] N. Lewis,[1] S. D. Patel,[1] S. Umezawa,[1] K. Watanabe,[3] T. Taniguchi,[4] and G. Moody*[1,2,b)]

[1)] *Electrical and Computer Engineering Department, University of California, Santa Barbara, CA 93106, USA*

[2)] *California Nanosystems Institute, University of California, Santa Barbara, CA 93106, USA*

[3)] *Research Center for Functional Materials, National Institute for Materials Science, 1-1 Namiki, Tsukuba 305-0044, Japan*

[4)] *International Center for Materials Nanoarchitectures, National Institute for Materials Science, 1-1 Namiki, Tsukuba 305-0044, Japan*

[a)] These authors contributed equally to this work.

[b)] Electronic mail: moody@ucsb.edu

NITROGEN-RICH SILICON NITRIDE DEPOSITION AND ANNEALING:

The nitrogen-rich SiN platform was designed to achieve negligible background emission after undergoing a high temperature rapid thermal anneal process, which is used for activating hBN defects in our study. 100 nm nitrogen-rich SiN films were grown on 3 μm thick thermal oxide substrates. All films were grown using a vision 310 advanced vacuum PECVD system at 300 C° and 800 mTorr with iterative 30 W, 13.56 MHz and 110 W, 187 kHz plasma steps for 8 and 1.5 seconds, respectively. The silane, ammonia, and nitrogen precursor flow rates were chosen to be 360 sccm, 18 sccm, and 980 sccm, respectively, to achieve nitrogen-rich growth conditions that quenches the SiN background emission while maintaining a high refractive index. Figure 2a of the main manuscript shows the measured refractive index of SiN for R = 29 (stoichiometric) to R = 5. With decreasing R, the refractive index decreases from 2.01 to 1.86 at 500 nm. We find that at R = 25, the background fluorescence begins to quench, as shown in Fig. 2b (main manuscript). Because of the trade-off between the reduction of the refractive index and the fluorescence quenching with decreasing R, we find that a ratio of R = 20 provides sufficient background-free SiN with only a moderate reduction of the index. By fine-tuning the PECVD parameters, it is possible to simultaneously achieve quenching while maintaining a refractive index > 1.9 without degrading the optical quality of the embedded 2D materials.

A 20-minute, 1000 ◦C rapid thermal anneal was performed to create optically active defects in hBN that was first transferred onto the Si3N4. Figure 1 shows the nitrogen-rich SiN films before and after a 20 minute 1000 C° rapid thermal anneal process with a 97 °C/minute ramp rate. The annealing chamber was conditioned with nitrogen and oxygen gas at flow rates of 100 sccm in order to minimize the background emission. Notably, we observed that non-stoichiometric films that were grown in more nitrogen-rich ratios suffered less from background emission activation during the annealing process. As seen in Fig.1, for the silane-to-ammonia ratio $R < 15$, the background emission is significantly suppressed compared to the near-stoichiometric recipe ($R = 25$). Note that the ability to anneal the SiN thin film without enabling background emission is crucial, as it allows the hBN emitters to be activated directly on the SiN film without the use of foreign anneal substrates and additional transfer steps[1].

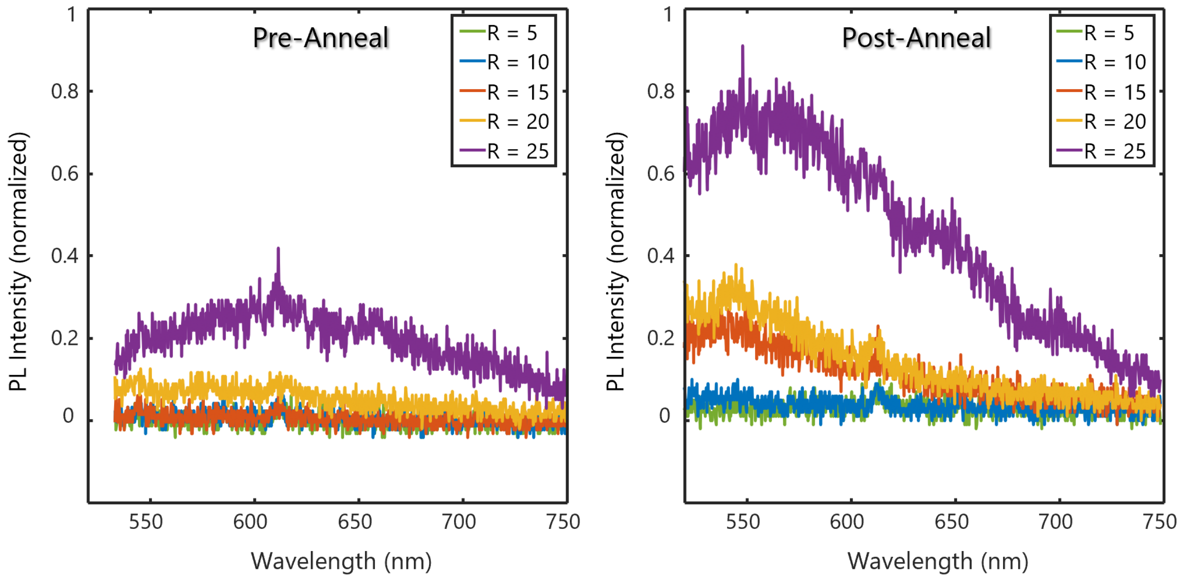

FIG. 1. **SiN pre- and post-rapid thermal annealing at 1000 C°.**

SIMULATIONS OF EMITTER-WAVEGUIDE COUPLING EFFICIENCY AND PHOTONIC COMPONENTS:

All simulations have been performed using Ansys Lumerical. Emitter-waveguide coupling efficiency has been calculated using Lumerical FDTD using a dipole source as an emitter. Coupling factor to the fundamental mode is calculated using a mode expansion monitor. Ring quality factor and free spectral range are simulated using Lumerical varFDTD with the fundamental TE mode source as an excitation. The mode volume of the cavity mode is calculated with FDTD using the formula $V = \frac{\int \varepsilon E^2 dv}{max(\varepsilon E^2)}$. Top collection efficiency of the photoluminescence using high NA objective (0.95) are performed using Lumerical FDTD through the integration over the acceptance cone. More details on the simulations can be found in the supplementary information.

A. Emitter-waveguide coupling efficiency

All of our waveguide and resonator cross-sections were designed for single-mode mode operation at the emission wavelength of the hBN single-quantum emitters (SQEs). Using the refractive index of SiN from Fig. 2(a) in the main text, the waveguide dimensions that support a single quasi-TE mode at wavelength $\sim$ 600 nm was found to be 650 nm in width with a fixed height of 100 nm. As mentioned in the main text, we explored multiple scenarios for placing the hBN emitters with respect to the waveguide. The coupling efficiency of a dipole source to the fundamental quasi-TE mode of the waveguide is simulated using Lumerical FDTD with the use of a mode expansion monitor that analyzes the fraction of power transmitted into any chosen mode. Results of the coupling coefficient (β) are shown in Fig.3(c) of the main text.

B. Edge coupler

We used an inverse taper as a spot size converter to expand the waveguide mode to match that of the fiber. The coupling efficiency of the fundamental TE mode to a fiber as a function of the taper length with a mode field diameter of 4.5μm calculated using Lumerical FDTD is shown in Fig. 2. Edge tapers are fabricated to be 125 μm long with an end cross-section of 110 nm width to ensure adiabatic mode transition with as little loss as possible, resulting in a simulated coupling efficiency of 66% at the SQE emission wavelength.

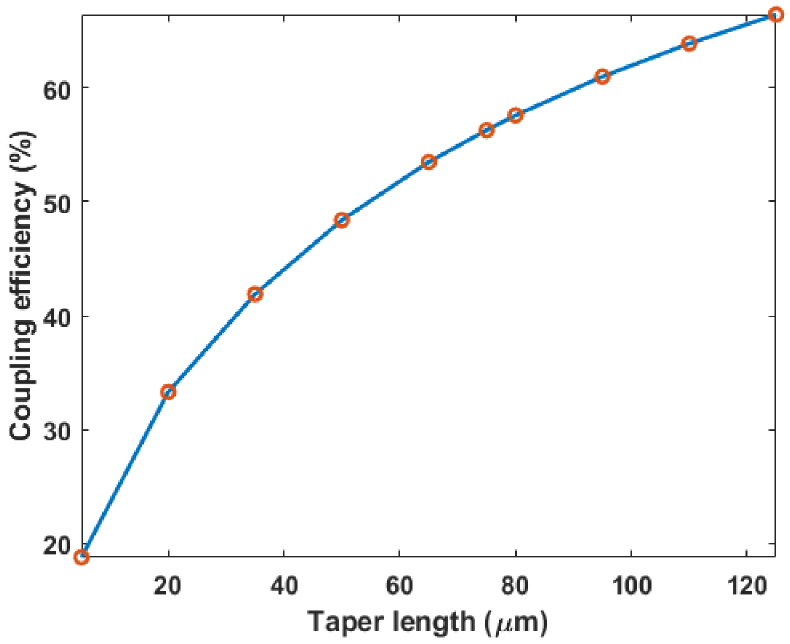

FIG. 2. Inverse taper coupling efficiency as the taper length is changed. The taper end cross section is 110 nm in width and 100 nm in height.

C. Coupling efficiency vs misalignment

Lumerical simulations, as discussed previously, were also used to estimate the coupling efficiency of the emitter to single mode waveguides as a function of misalignment for both embedded-hBN and bottom-hBN configurations. Figure 3 demonstrates as the dipole is moved away from the center of the waveguide, the efficiency decreases as expected. Similar results have also been reported earlier in the literature. The decrease in efficiency for the first 100 nm of misalignment is about 10%, which demonstrates that the accuracy of our sub-100 nm integration method is sufficient to place the emitters in excellent overlap with the optical cavity mode.

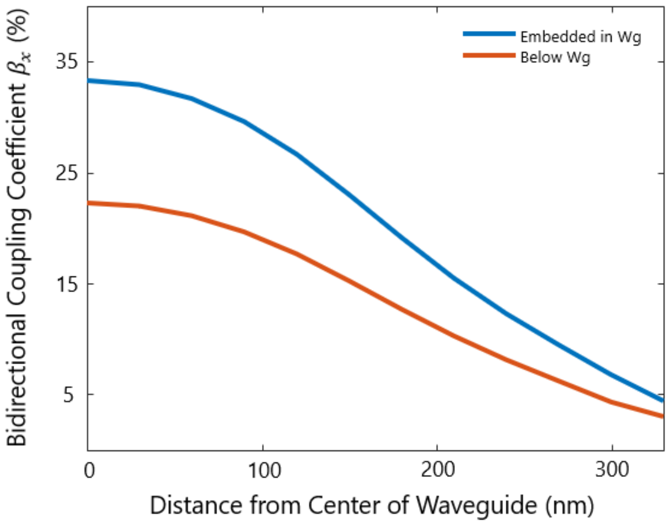

FIG. 3. Emitter-waveguide coupling efficiency as a function of emitter misalignment from the center of the waveguide.

OPTICAL SETUP:

Figure 4 is a schematic illustration of the optical setup used in the experiments. The main laser source is a continuous-wave 532 nm green laser diode. A dichroic mirror at 540 nm is used to separate excitation and collection paths. In addition, a 600 nm long-pass spectral filter was used to further extinguish the excitation laser in the collection path. A second dichroic mirror at 510 nm is used to separate the camera and white light path from the excitation/collection path. The use of a second dichroic not only doubles the signal collection efficiency from the sample, but it is also necessary to minimize the chromatic aberrations in the optical images that are used for the alignment process in this study. An infinity-corrected 0.9 NA dry objective with 1 mm working distance is used for spectroscopy. For the edge coupling, a low profile (1.4 mm total height) customized v-groove array (4x PM460-HP OZ Optics) is used. The low profile v-groove fiber array enables us to collect light from the edge while simultaneously exciting the SQE with the 0.9 NA objective. To characterize the microring resonator transmission spectrum, a superluminescent diode (Thorlabs SLD635T) was coupled on and off chip using a two-channel single-mode v-groove PM460-HP fiber array.

Second-order autocorrelation measurements with continuous-wave excitation were performed by passing the collected emission through the side-slit of the spectrometer (Princeton instruments HRS-500) using a 300 groove/mm grating set at the center ZPL wavelength. The filtered light was sent into a multimode fiber beamsplitter and fed into two single-photon avalanche detectors (Excelitas SPCM-AQRH-13-FC) through two optical circulators to minimize optical cross-talk between the detectors. The electrical signals from detectors (350 ps timing resolution) were analyzed using a Swabian Time Tagger Ultra photon counting module (8 ps timing resolution).

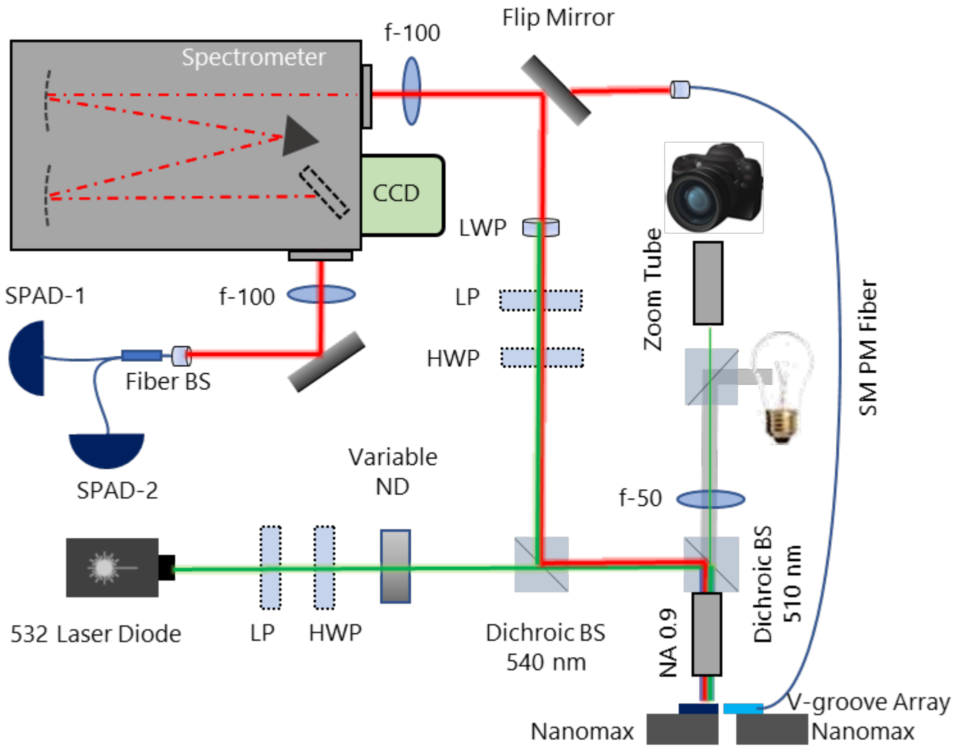

FIG. 4. **Optical Setup Schematic**

PHOTONIC FABRICATION AND DEFECT ALIGNMENT PROCESS:

The fabrication process for integrating 2D quantum emitters within SiN photonic structures is illustrated in Fig. 2e-2j of the main manuscript and is described in more details here. Briefly, SiN thin films were grown by PECVD on a 3-μm-thick SiO_2 film on silicon, where the film thickness is determined by the desired position of the flake in the waveguide structure normal to the sample plane. A variety of SiN films were grown with an increasing ammonia-to-silane ratio to characterize the 2D material transfer, annealing, and full fabrication process. Using an all-dry visco-elastic transfer technique, 2D material flakes of hBN or WS_2 are transferred within the electron-beam processing window of 200 $\times$ 200 μm. For hBN flakes, a 5-minute, 250 watt O_2 plasma and rapid thermal annealing step at 1000 °C activates the quantum emitters, which are then pre-screened by scanning the sample in a 0.9 numerical-aperture (NA) photoluminescence microscopy setup. The emission brightness and single-photon purity are the primary metrics we use to identify suitable emitters for integration. Once the target emitters and flakes are identified, to align the waveguide mask to the SQEs, first horizontal and vertical fine alignment metal bar array structures (500 nm spaced bars) are patterned in a location roughly 20 μm away from the flake using electron-beam lithography (Nabity NPGS SEM) and standard lift-off techniques (PMMA) (Fig.5(a-b)).

To visually identify the thin flake under PMMA during the alignment process, a small identification scratch mark is created in the PMMA using an electrical contact probe. This allows us to align the center field of the pattern to our desired location within a few micrometer error margin, which is sufficient to obtain the flake and array structures within the same field of view of our imaging system. Next, in the photoluminescence (PL) spectroscopy setup, a 532 nm green laser is manually scanned to find the targeted emitter. After finding the emitter, an open-loop piezo-controlled positioner (Thorlabs NanoMax MAX312D) is scanned with maximum resolution ($\sim$50 nm) to find the maximum intensity point of the defect. The accuracy can be increased in the future by using closed-loop piezo stages or scanners.

Next, within the same setup, using a blue light microscope assembly (infinity corrected 100x objective with 0.9 NA 1 mm working distance with an f = 50 achromatic doublet, imaged through a zoom lens) the optical images of the flakes are taken when the laser spot is centered at the location of the SQE and the alignment marks are in the field of view (FOV, 70 μm x 70 μm), as shown in Fig.6(a). The optical images are then processed in Matlab using an edge detection code

based on the "canny edge detection" technique. First, the edges of the fine-alignment bars are detected. Then the image scale undergoes a matrix transformation until the mean square error of the difference between the actual and the optically detected patterns are minimized. Afterward, the corrected image is overlaid with the pattern in K-Layout software and the location of the center of the laser spot with respect to the center of the pattern is determined. The pattern of the ring is then aligned to the image based on the measured emission dipole of the defect, as illustrated in Fig.6(b). The initial spacing of the fine-alignment metal bars were designed so that at least 2 μm spacing would exist between the alignment marks and the microring, ensuring no waveguide propagation loss due to the metal. The key step of this process is using both X and Y alignment bar arrays that are visible the FOV of the microscope. This allows for a sufficient amount of identified edges along both the X- and Y-axis that can be used to correct for any image distortion.

Next, a reactive-ion etching step is utilized to define the photonic structures. An optional $0.1 \sim 1$ μm PECVD SiO_2 cladding layer is grown to shield the structures from the environment. Finally, the chip is diced at the waveguide taper region to provide optical access.

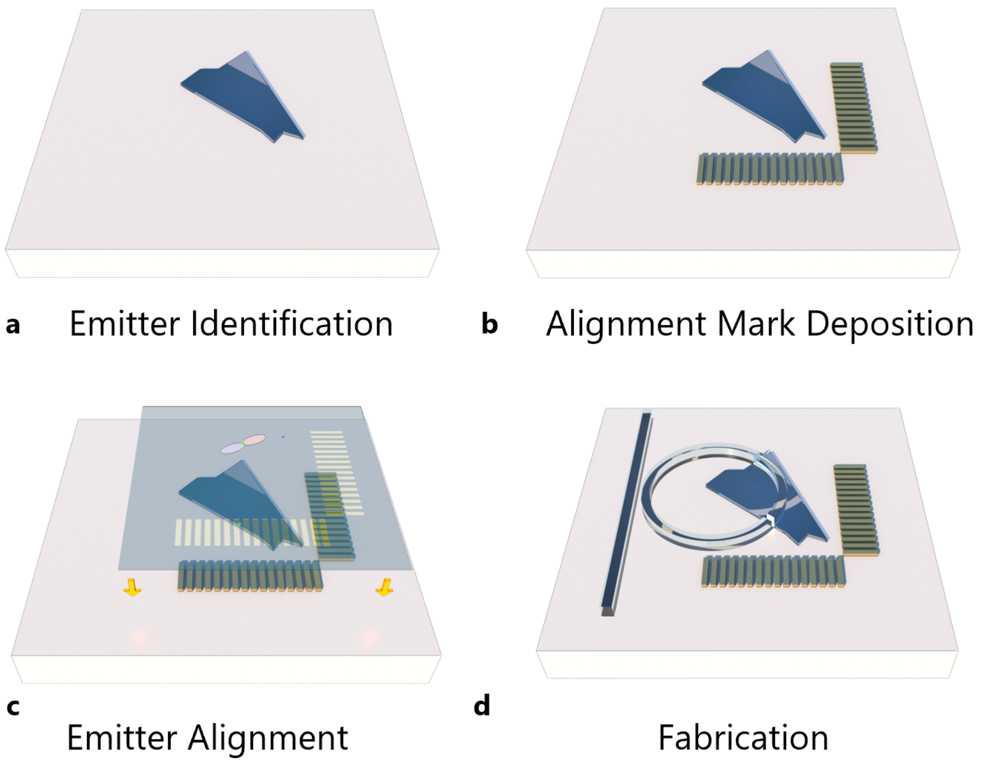

FIG. 5. **3D schematics of the alignment process** **a**, Emitters are initially identified through PL spectroscopy. **b**, Fine alignment-bar arrays are deposited next to the flake. **c** emitter is re-identified in PL spectroscopy. The laser is centered at the SQE location, and using blue-light imaging, optical images of the flake are taken when the alignment bars are in the microscope FOV. The image undergoes correction using identified edges of the alignment bars and the initial pattern as a reference. The ring pattern is centered on the defect based on the emission dipole of the defect. **d** Final fabricated device.

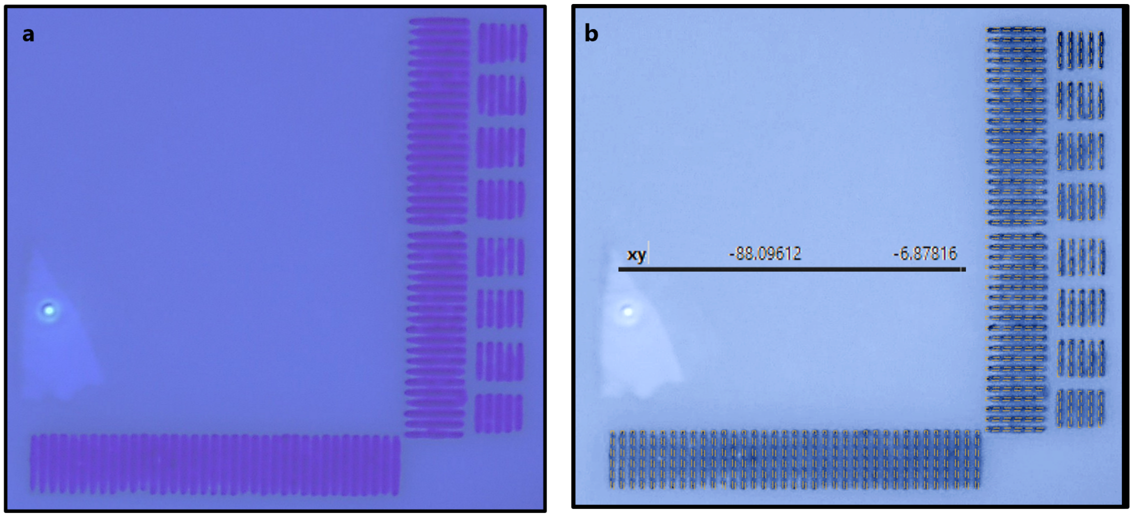

FIG. 6. **Optical images of the alignment process a**, Optical image during the re-identification process. The SQE location is be denoted by the green laser spot. Note that the laser is attenuated using an additional notch-filter while the blue-light imaging is achieved by passing a white light source through a dichroic mirror. **b**, Processed image with corrections. The reference pattern of the alignment marks (dashed orange lines) are now overlaid with the alignment bars. The location of the defect with respect to the center of the first pattern is read and displayed on the image.

MICRORESONATOR CHARACTERIZATION:

A superluminescent diode (SLED, 638 nm center wavelength) was used to characterize the transmission spectrum of the microresonators. The SLED emission is first coupled into a single-mode v-groove fiber array (4-PM-460HP with 127 μm spacing) and then launched into the exposed facets of the tapered waveguides. The second port of the waveguide is then used to read out the response of the photonic chip and is sent to the spectrometer. Figure 7(a) shows an optical image of the device under test. The normalized spectrum of the SLED is de-convolved from the output spectrum (Fig.7(b)) to yield the normalized transmission spectrum of the microresonator.

The normalized transmission power through a microresonator can be represented as:

$$\frac{|B|^2}{|A|^2} = \frac{t^2 - 2t|\eta|\cos(\phi) + |\eta|^2}{1 - 2t|\eta|\cos(\phi) + |\eta|^2 t^2}, \tag{1}$$

where $|B|^2$, $|A|^2$, t, η, and ϕ are the transmitted power, input power, transmission coefficient, round-trip loss coefficient, and round-trip phase, respectively. On resonance ($\cos(\phi) = 1$), the extinction coefficient ($|B|^2/|A|^2$) can be expressed as:

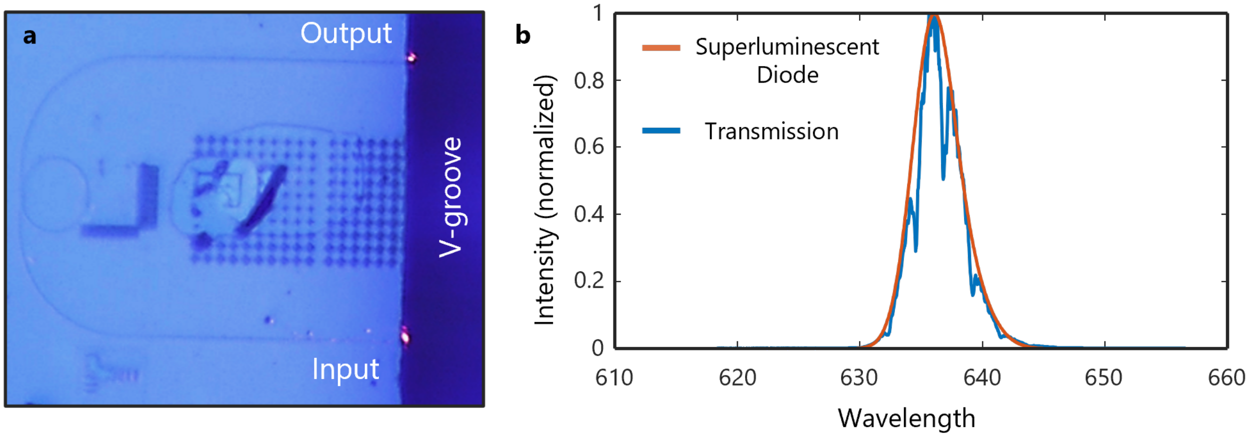

FIG. 7. **Microresonator characterization. a**, Optical image of a device under test. The input port is used to launch a broadband superluminescent diode into the waveguide. Light is collected from the output port. **b**, The transmission spectrum of the superluminescent diode collected from the top waveguide is indicated by the blue curve, and the raw spectrum of the superluminescent diode is shown by the orange curve for reference. Dips in the transmission correspond to the resonances of the microresonator. The normalized microresonator transmission and reference spectra are fit using the presented theoretical model to determine the microresonator response.

$$\varepsilon = \frac{|B|^2}{|A|^2} = \frac{(t-|\eta|)^2}{(1-t|\eta|)^2}. \tag{2}$$

Similarly, the quality factor can be expressed as:

$$Q = \frac{\pi n_g 2\pi R}{\lambda} \frac{\sqrt{t|\eta|}}{\lambda(1-t|\eta|)}, \tag{3}$$

where n_g, R, and λ are the group index, radius of the ring, and the wavelength, respectively. Hence, by extracting the values of the extinction factor ε and Q of the resonances from the transmission spectrum, equations 1 and 2 can be used to solve directly for t and η. Knowing t and η, the coupling coefficient ($\kappa = 1-t^2$) and the intrinsic loss per meter α can be calculated. Then, the coupling quality factor (Q_c) and the intrinsic quality factor (Q_i) can be expressed as:

$$Q_c = \frac{\omega\tau_{rt}}{1-t^2} \tag{4}$$

$$Q_i = \frac{\omega\tau_{rt}}{2\pi R\alpha}, \tag{5}$$

where the τ_{rt} is the round-trip time in the ring.

Equation 2 has two distinct solutions depending on whether $t > |\eta|$ (under-coupled condition) or $|\eta| > t$ (over-coupled condition). This yields two sets of different Q_c and Q_i depending on the over/under-coupled condition for which the system is solved. Hence, first, it is imperative to establish whether the system is in the under-coupled or over-coupled condition.

To establish a baseline for our microresonators, we fit the data of over twenty samples in three fabrication runs. From our Lumerical simulations, for our designed race-track lengths of 3 μm, a κ^2 value of 0.18 is extracted at the 635 nm wavelength. Figure 8(a) presents the average extracted κ values for our rings under both over-coupled (red) and under-coupled (black) conditions. The under-coupled condition matches the Lumerical simulations closely, supporting our assessment that all of the fabricated devices are operating in the under-coupled regime. Figure 8(b) represents the extracted Q_c and Q_i of over 10 measured devices for the microring with 3 μm coupling region length, which is the same configuration for which we fabricated the integrated devices.

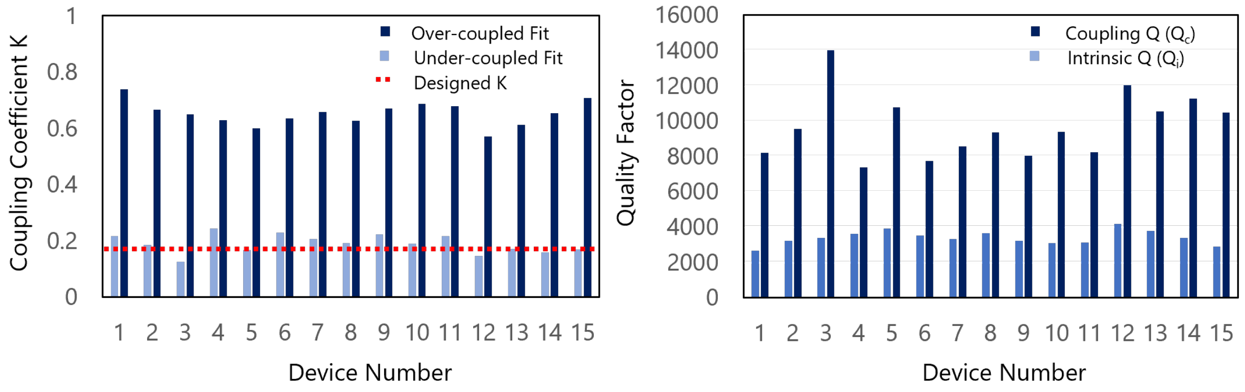

FIG. 8. **Fit to the theoretical model. a**, Coupling coefficient (K) extracted from fitting the normalized transmission plots with under-coupled and over-coupled assumptions of the theoretical model. The under-coupled fit closely matches the designed coupling coefficient extracted from the Lumerical simulations. **b**, Extracted coupling and intrinsic quality factors with the under-coupled assumption. The coupling quality factor average is $Q_c = 9690$ with a standard deviation of 1834. The intrinsic quality factor average is $Q_i = 3560$ with standard deviation of 393.

SYSTEM EFFICIENCY ESTIMATIONS

Wave-guide facet loss was calculated by launching a superluminsecent diode at 638 nm into the input port of the waveguide (Fig.7a) and measuring the spectral intensity and power at the output port. An average facet loss of 17.5 db per facet was measured. The rest of the side collection path efficiency was similarly quantified by measuring the initial intensity of a fiber coupled red-laser and measuring the total power at the slit of the spectrometer. Top collection path was quantified by reflecting a red laser off of a mirror at the sample stage and measuring the overall transmitted power up to the slit.

To estimate the objective collection efficiency, We use Lumerical FDTD to estimate the percentage of dipole emission upwards towards the objective. The far field projection is integrated over a cone that corresponds to the objective's NA. The simulations are performed with a dipole source embedded underneath the SiN with a 3 μm-thick SiO_2 cladding layer. We used two ways for integrating the power in the far field of the structure. The first method integrates the electric field intensity over the objective's acceptance angle and then normalizes the result to the total transmitted power of the dipole emitter going through the monitor. The second method integrates the Poynting vector in the far field over the solid angle and then normalizes it to the total power emitted from the dipole source. For 610 nm wavelength, the collection efficiency of the objective is 10.3% and 13.2% using method 1 and method 2, respectively. For 750 nm wavelength, the calculations result in 13.3% and 13.8% of the light captured by the objective from the two calculation methods.

Figure 9 represents coupling efficiency and Purcell factor estimation for both hBN SQE and WS_2 integrated devices. The blue region in each panel demonstrates the standard deviation error bound for Q_c extraction illustrated in Fig.8(b), and the red region represents the minimum and maximum estimations for objective extraction efficiency as discussed above. The reported values in the manuscript are taken from the error bounds at the average collection efficiency of the two methods, resulting in 11.8% for hBN and 13.5% for WS_2.

Finally, we emphasize that, in this study, our focus was on optimizing β, it is also equally important to maximize η_{out}, which can be expressed as the ratio Q/Q_c with Q and Q_c defined as the loaded and coupling quality factors of the cavity. For the cavity-coupled system shown in the manuscript, η_{out} is estimated to be 7% of the total coupled light into the cavity, leading to total system efficiency of 2.8%. This is a direct result of the resonator being under-coupled

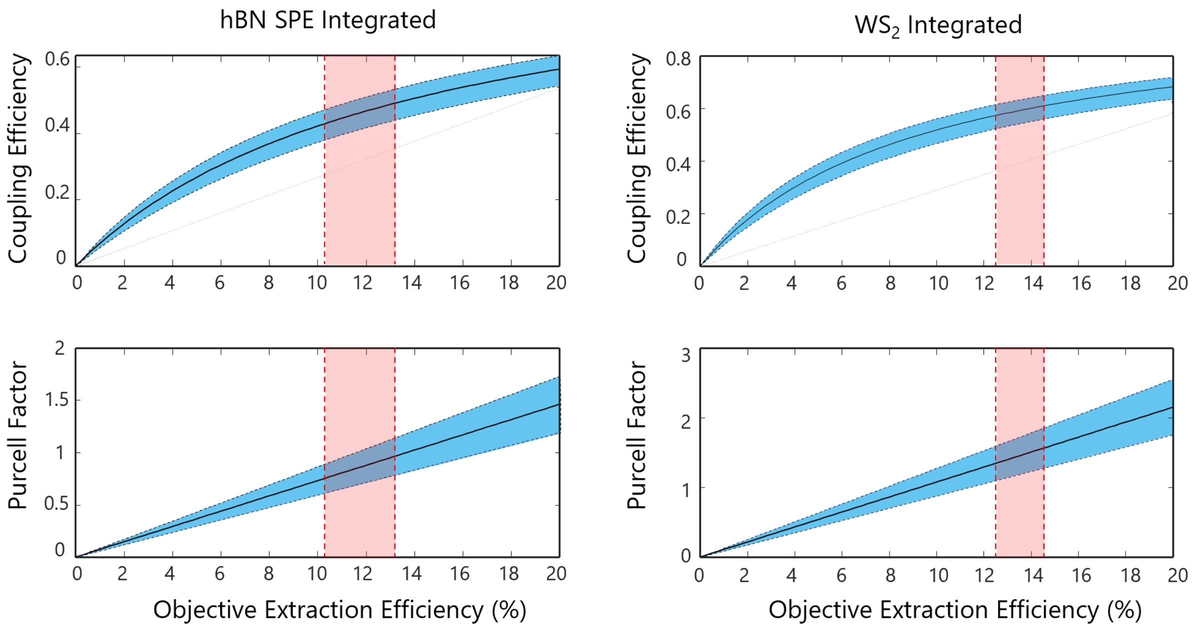

FIG. 9. **Coupling efficiency and Purcell factor estimation as a function of objective collection efficiency.** The error bound for WS_2 and hBN has been extracted from the standard deviation in the Q_c values. The red region represents the estimated objective extraction efficiencies using Lumerical for hBN (610 nm) and WS_2 (640 nm).

($Q_c > Q_i$). By extending the waveguide-cavity coupler, Q_c can be decreased to ~3000 with only a 14% reduction of the loaded quality factor and the Purcell factor. This would result in a significant enhancement of the cavity out-coupling efficiency to $\eta_{out} > 17\%$ and the total system efficiency to 7%.

PROJECTING THE PERFORMANCE OF 2D EMITTERS USING JAYNES-CUMMINGS MODEL:

We follow the solutions to the master equations for a dielectric nanophotonic waveguide that evanescently interacts with a cavity-emitter system, similar to the framework previously developed[2], but with modifications for emitter-cavity coupling. Assuming cryogenic temperature, under the assumption that the pure dephasing rate is negligible compared to the total emitter and cavity decay rates, it can be shown that the probability of an excited emitter generating a photon into the waveguide mode can be represented as:

$$\eta = \frac{\kappa}{(\gamma_e + \gamma_p)(1 + \frac{\gamma_e \gamma_p}{4\Omega^2})}. \tag{6}$$

where κ, γ_e, γ_p, and Ω are the cavity coupling into the bus waveguide, the emitter linewidth, total cavity decay rate including the intrinsic loss and cavity-bus coupling, and the cavity-emitter coupling strength. The rates can be related to quality factors via $\gamma_c = \frac{\omega_c}{2Q_i}$, and $\kappa = \frac{\omega_c}{2Q_k}$. Furthermore, Ω is given by:

$$\Omega = \sqrt{\frac{3\pi c^3}{2n_d\omega_c^2} \cdot cos^2\theta_d \left(\frac{\Gamma}{V_c}\right)}, \tag{7}$$

where ω_c, n_d, Γ, θ_d, and V_c are the resonant frequency of the cavity, refractive index of the 2D material, the emitter radiative decay rate, angle between the emitter polarization axis and the cavity field polarization, and the cavity mode volume. To capture the dynamics of the system close to the bad-emitter regime, we have applied a heuristic correction factor ζ to the cavity-emitter coupling rate (Ω), expressed as:

$$\zeta = \frac{\kappa + \gamma_c}{\kappa + \gamma_c + \Gamma}, \tag{8}$$

which accounts for partial coupling of the emitter into the cavity due to spectral mismatch[3].

To determine the photon extraction efficiency given by Eqn. 6 as a function of the loaded quality factor of the cavity, $Q = \left(\frac{1}{Q_i} + \frac{1}{Q_k}\right)^{-1}$, we sweep Q_k with Q_i remaining at a constant value of 10^8. The dependence of η on cavity volume V_c is also swept and contributes to Ω. In addition, we set $\theta_d = 1$ because our defect positioning and alignment process ensures near-ideal placement and orientation between the electric field and polarization axis of the mode. Lastly, the emitter quality factor is assumed to be of a similar form to that of the intrinsic cavity decay and coupling rates, i.e. $Q_e = \frac{\omega_c}{2\gamma_e}$. The total emitter decay rate, $\Gamma = \gamma_e \cdot \eta_Q$ is obtained as a product of the emitter

decay rate and its quantum efficiency (assuming no additional pure dephasing). For various 2D emitter properties, the η dependence on the quality factor and the normalized cavity volume is shown in Fig. 10. The parameters used in the simulations in the figure are shown in Table 1 and are extracted from references[4,5].

Material	λ_0 (nm)	τ (ns)	γ^* (MHz)	η_Q	n_d	Q_i
hBN	600	1.2	150	0.87	2.1	10^8
WSe_2	800	10	2000	0.05	4.2	10^8

TABLE I. Material properties and parameters used in the calculation of single photon extraction efficiency as given by eqn. 6 as a function of the cavity's loaded quality factor, and the cavity volume normalized by the modal volume. Where λ_0 represents the free-space wavelength of the emitter, τ represents the emitter lifetime, η_Q represents the quantum efficiency, n_d represents the material's refractive index, and Q_i representing the intrinsic quality factor.

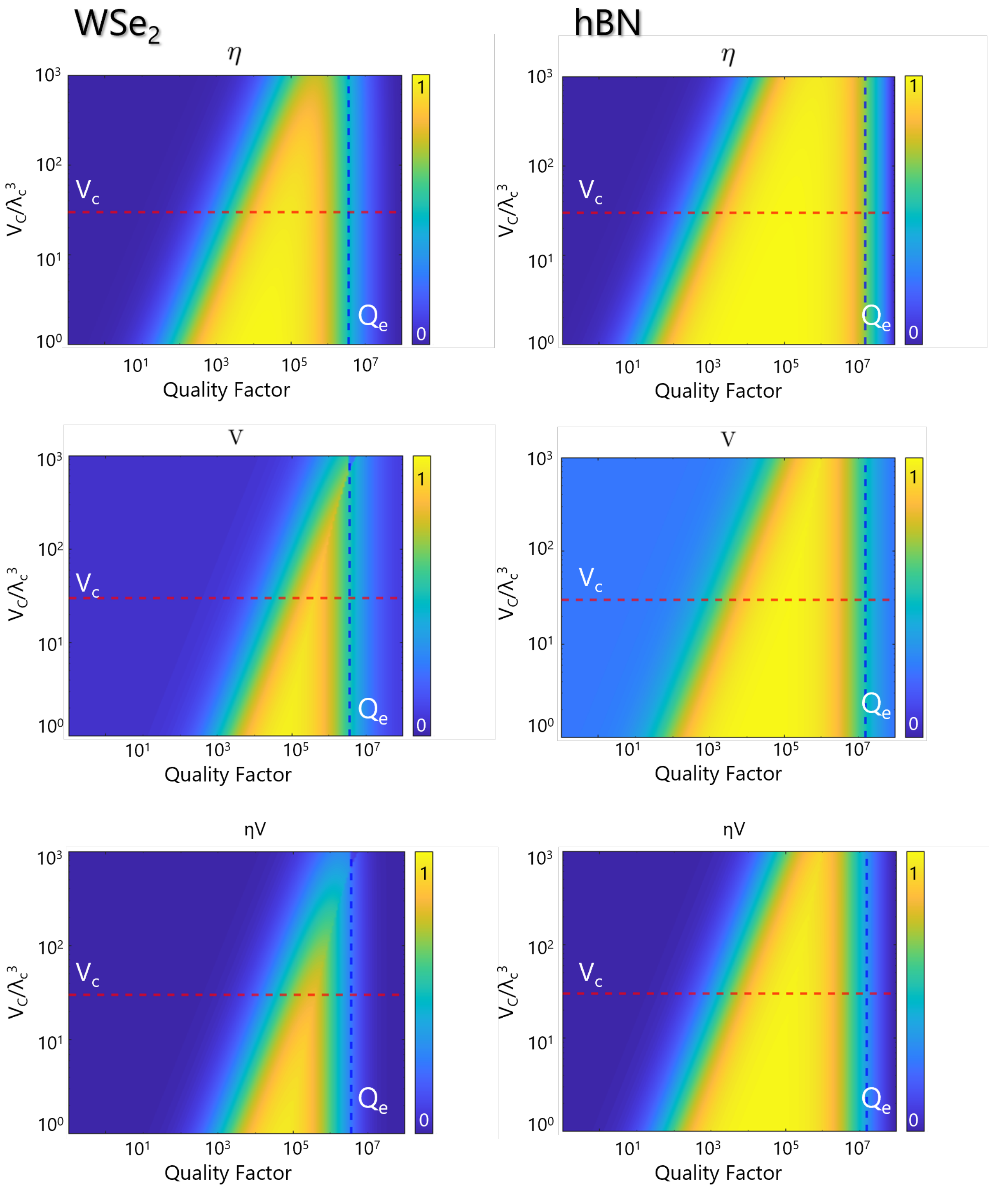

FIG. 10. **System efficiency η (emission of photons into the bus waveguide), blueindistinguishability (V), and system efficiency indistinguishability product ($\eta \times V$) as a function of the loaded quality factor of the cavity and the cavity volume normalized by the mode volume.** Contour plots based on Eqn. 6 rely on parameters listed in Table I.

SECOND-ORDER AUTO-CORRELATION BEFORE INTEGRATION:

Figure 11 presents the second-order auto-correlation function for the emitter prior to integration. We observe a 13% lifetime shortening after cavity integration. Note that for an emitter with quantum efficiency η_{qe} inside a cavity with a Purcell factor of 0.86, the measured reduction of the lifetime can be expressed as:

$$\frac{\tau_c}{\tau} = \frac{1}{1-\eta_{qe}+AF\eta_{qe}}, \tag{9}$$

where η_{qe}, F, and A are the quantum efficiency, Purcell factor, and the overlap ratio of the ZPL with the cavity resonances, respectively. For our emitters, assuming a quantum efficiency as high as 0.8 and assuming the fabrication process does not introduce any additional non-radiative losses, we would expect a lifetime reduction about 25%, which is consistent with our measured lifetime; however, care must be taken in interpreting and analyzing lifetime data, since to extract Purcell factors from these measurements, it is required to make assumptions about the emitter's quantum efficiency and modifications to the surrounding dielectric environment, which are challenging to establish and verify.

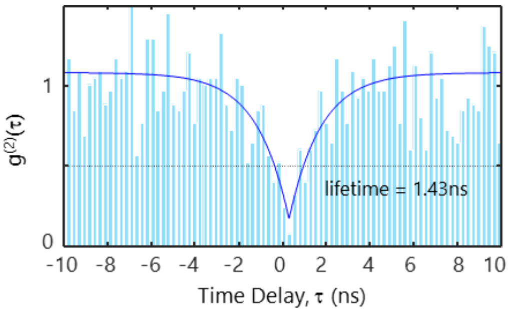

FIG. 11. **Second-order auto-correlation of hBN emitter before integration. The measured 1.43 ns lifetime is reduced to 1.23 ns after integration.**

REFERENCES

[1] A. W. Elshaari, A. Skalli, S. Gyger, M. Nurizzo, L. Schweickert, I. Esmaeil Zadeh, M. Svedendahl, S. Steinhauer, and V. Zwiller, "Deterministic integration of hBN emitter in silicon nitride photonic waveguide," Advanced Quantum Technologies **4**, 2100032 (2021).

[2]F. Peyskens, C. Chakraborty, M. Muneeb, D. Van Thourhout, and D. Englund, "Integration of single photon emitters in 2D layered materials with a silicon nitride photonic chip," Nature Communications **10**, 4435 (2019).

[3]H. Kaupp, C. Deutsch, H.-C. Chang, J. Reichel, T. W. Hänsch, and D. Hunger, "Scaling laws of the cavity enhancement for nitrogen-vacancy centers in diamond," Physical Review A **88**, 053812 (2013).

[4]K. Parto, S. I. Azzam, K. Banerjee, and G. Moody, "Defect and strain engineering of monolayer WSe_2 enables site-controlled single-photon emission up to 150 K," Nature Communications **12**, 3585 (2021).

[5]N. Nikolay, N. Mendelson, E. Özelci, B. Sontheimer, F. Böhm, G. Kewes, M. Toth, I. Aharonovich, and O. Benson, "Direct measurement of quantum efficiency of single-photon emitters in hexagonal boron nitride," Optica **6**, 1084–1088 (2019).
